# *In Vitro* Seeds Germination and Seedling Growth of Bambara Groundnut (*Vigna subterranea* (L.) Verdc. (Fabaceae)) 

**DOI:** 10.1155/2015/595073

**Published:** 2015-10-13

**Authors:** Mongomaké Koné, Tchoa Koné, Nakpalo Silué, André Brahima Soumahoro, Tanoh Hilaire Kouakou

**Affiliations:** ^1^Laboratoire de Biologie et Amélioration des Productions Végétales, UFR des Sciences de la Nature, Université Nangui Abrogoua, 02 BP 801, Abidjan 02, Côte d'Ivoire; ^2^Laboratoire de Physiologie Végétale, UFR Biosciences, Université Félix Houphouët-Boigny, 22 BP 582, Abidjan 22, Côte d'Ivoire

## Abstract

Bambara groundnut (*Vigna subterranea* (L.) Verdc.) is an indigenous grain legume. It occupies a prominent place in the strategies to ensure food security in sub-Saharan Africa. Development of an efficient *in vitro* regeneration system, a prerequisite for genetic transformation application, requires the establishment of optimal conditions for seeds germination and plantlets development. Three types of seeds were inoculated on different basal media devoid of growth regulators. Various strengths of the medium of choice and the type and concentration of carbon source were also investigated. Responses to germination varied with the type of seed. Embryonic axis (EA) followed by seeds without coat (SWtC) germinated rapidly and expressed a high rate of germination. The growth performances of plantlets varied with the basal medium composition and the seeds type. The optimal growth performances of plants were displayed on half strength MS basal medium with SWtC and EA as source of seeds. Addition of 3% sucrose in the culture medium was more suitable for a maximum growth of plantlets derived from EA.

## 1. Introduction

Bambara groundnut (*Vigna subterranea* (L.) Verdc.) plays an important role in reducing food insecurity and child malnutrition and improving the economic power of households. Ranked among the minor species, the plant has risen in recent decades, a renewed interest among research funders due to its ability to contribute to the diversification of food crops in Africa [1, 2]. As a leguminous plant, Bambara groundnut is useful in crop rotations as a source of residue nitrogen for the subsequent crop through nitrogen fixation [3].

Despite its importance, Bambara groundnut cultivation is limited by its sensitivity to plant pathogens and low and unpredictable yields (650–850 kg·ha^−1^) for the majority of semi-Arid Tropics [4]. Furthermore, the presence of antinutritional factors in the seeds, such as tannins and trypsin inhibitors [5], limits digestion and availability of nutrients from the seed.

The popularization of Bambara necessarily involves intensification of its production and in particular the creation of improved varieties to overcome the numerous cultural constraints of the plant. Improvement of Bambara groundnut can be achieved by genetic recombination and selection, but reports in this domain are limited [6]. Artificial hybridization is extremely difficult and very low success rates have been reported (<2% harvested hybrid seeds) [2]. Moreover, conventional breeding methods are time consuming and laborious.

Recent advances in biotechnology including the direct integration of genes into plants have offered the opportunity to develop improved germplasm by overcoming the difficulties to cross distant taxa. The development of such technologies largely depends on the development of efficient plant regeneration protocols. Before achieving this goal, the availability of healthy explants derived from* in vitro* seedling is a prerequisite. Therefore,* in vitro* germination of seeds was attempted in order to obtain young and surface sterile plant material for the subsequent establishment of* in vitro* propagation protocols, necessary for the intended exploitation of this indigenous plant through biotechnological means. Seedlings obtained* in vitro* are of great importance since they are directly used for other tissue culture experiments without any sterilization.* In vitro* tissue culture is important to offer high rates of multiplication from segments of tissue; however, it is also an efficient tool for obtaining large numbers of individuals free of contaminating sources [7–9].

Protocols for* in vitro* germination of seeds and seedling growth have been established in many leguminous species including* Bobgunnia madagascariensis* (Desv.) J. H. Kirkbr. & Wiersema [10],* Pterocarpus marsupium* Roxb. [11], and* Cordeauxia edulis* Hemsl. [12]. Different basal media [10], salts strength [13], type and concentration of plant growth regulators [14], and mature and immature seed [15] have been used for germination and propagation.

In Bambara groundnut, seeds germination and the development of* in vitro* plantlets using only one basal medium (BM) have been reported [16, 17]. Similarly,* in vitro* germination of seed for obtaining* in vitro* plantlets of Bambara was carried out in our previous works [18–20]. However, assessment of this process has never been undertaken. Herein, the purpose of this research was to establish optimal conditions for the seeds germination and* in vitro* seedling growth of Bambara groundnut based on the responses to the culture media composition and the seeds types used as explants. The results from this study can be used for plant regeneration of different Bambara groundnut landraces.

## 2. Materials and Methods

### 2.1. Seed Sources and Surface Sterilization

**Figure 1:**
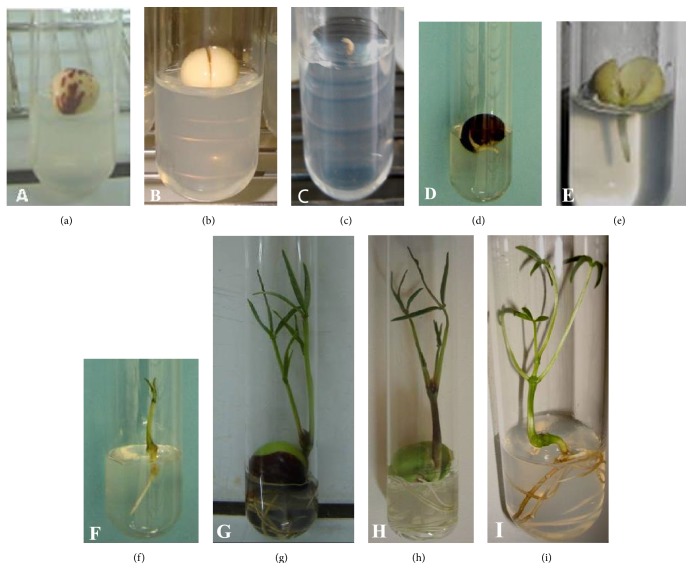
*In vitro* seeds germination and seedling development of* Bambara groundnut*. ((a)–(c)) Seeds explants type on MS medium containing 3% sucrose. ((d)–(f)) Cultured seeds type on MS medium containing 3% sucrose after five days of cultivation. ((g)–(i)) Developed seedling derived from seed with seed coat (g); seed without seed coat (h); and embryonic axis (i) after three weeks of incubation on MS medium containing 3% sucrose.

The mature seeds of Bambara groundnut (cv. Ci2) were collected from the plants grown on experimental plots in University Nangui Abrogoua. The seeds were stored in air tight plastic bottles at room temperature. Under a laminar airflow cabinet, the seeds were surface-sterilized sequentially with 70% (v/v) ethyl alcohol (30 sec.) and 7% (w/v) calcium hypochlorite solution (30 min) and finally rinsed thoroughly three times. The seeds were soaked overnight in 100 mL beaker containing 50 mL of sterile distilled water (SDW). After soaking overnight, the water was discarded, the seeds were rinsed 3-4 times with SDW, and then three (03) types of seeds were used for* in vitro* germination trials. (i) Seeds with seed coat (SWC) (Figure 1(a)), (ii) seeds without seed coats (SWtC) (Figure 1(b)), and (iii) the embryonic axis carefully isolated after the cotyledons have been separated (Figure 1(c)).

### 2.2. Effect of Different Basal Media

The three types of seeds were cultured on five (5) basal media, namely, MS [21], B5 [22], SH [23], MC [24], and Chu N^6^ (CHU) [25]. 3% sucrose was added to these basal media containing no growth regulators. The pH of the culture media was adjusted to 5.8 before adding 0.6% (w/v) agar. Culture medium without any fortification in nutrients served as control.

### 2.3. Effect of Medium Strength

To optimize germination and the* in vitro* development of plantlets, the seeds types were inoculated on three strengths of MS medium, namely, full strength MS salts (MS), half strength MS salts (1/2 MS), and quarter strength MS salts (1/4 MS).

### 2.4. Effect of Carbohydrate Sources

The best culture medium from the previous experiment was selected for testing different sources of carbohydrate. Thus, in this germination medium, 3% of sucrose, glucose, and fructose have been added, respectively, for their effectiveness in promoting the germination and the seedling subsequent growth.

### 2.5. Effect of Sugar Concentrations

Different concentrations (1, 2, 3, 4, 5, and 6%) of the best carbon source defined in the previous experiment were tested. Seeds were cultured on these culture media with a composition varying in sugar concentration.

### 2.6. Culture Conditions

All the culture media were autoclaved at 120°C for 20 min. For all the experiments described above, the cultures were transferred into a culture room, where they were maintained at 25 ± 2°C under 16/8 hours' (light/dark) photoperiod with light intensity of 3000 lux provided through white fluorescent tubes.

### 2.7. Data Collection on Germination and Growth Parameters

Germination was defined as the appearance of a 2 mm radicle and is referred to as physiological germination throughout this paper. Germination was monitored daily for each seed type until no further germination was recorded and the mean germination time (MGT) was calculated using the formula cited by [26] given below:

(1) $$\begin{array}{c} \text{MGT}=\frac{\sum \left(nT\right)}{\sum n}, \end{array}$$

where *n* is the number of seeds newly germinated at time *T* at 25°C; *T* represents hours from the beginning of the germination test; ∑*n* is the final germination.

The total numbers of the germinated seeds for each treatment were summed up to determine the cumulative germination and the rate of germination was calculated following the procedure of [27].

Four (4) weeks after sowing, seedlings height, epicotyl and primary root length, and the number of leaves, branches, and secondary roots were recorded. The dry weights of aerial and root systems were obtained by drying them in an oven at 65°C for 72 hours until a constant dry weight.

## 3. Statistical Analysis of Data

The experiment was carried out in a completely randomized design with ten replicates and each individual treatment was repeated three times. Data were submitted to analysis of variance (ANOVA) to detect significant differences between means. Means differing significantly were compared using Newman-Keuls multiple range test at the 5% probability level using statistical software program Statistica version 7.0.

## 4. Results and Discussion

### 4.1. Effect of Basal Medium on* In Vitro* Germination of Seeds and the Plantlets Development

**Figure 2:**
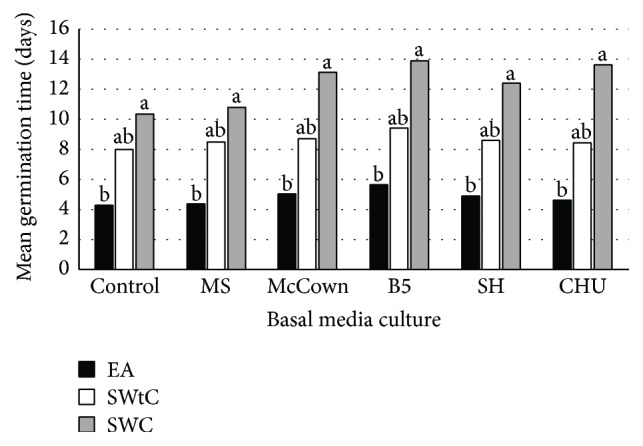
Mean germination time of different seeds type of Bambara groundnut after three weeks of incubation on different basal media. EA: embryonic axis; SWtC: seed without seed coat; SWC: seed with seed coat.

**Figure 3:**
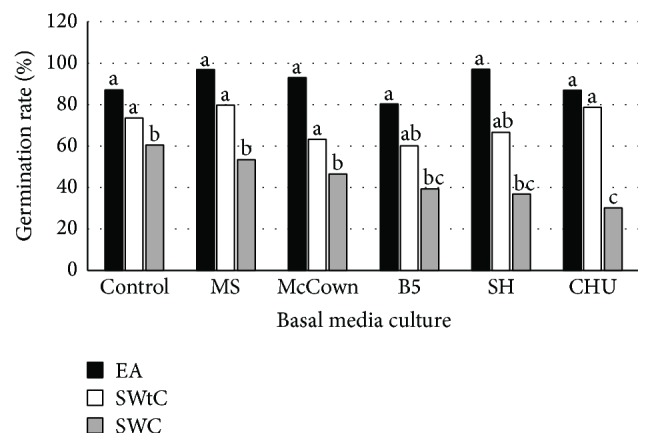
Germination rate of different seeds type of Bambara groundnut after three weeks of incubation on different basal media.

After incubation on the culture media, seeds became swollen quickly and germination occurred within the first two weeks of culture (Figures 1(d)–1(i)). The mean germination time (MGT) and the mean germination rate (MGR) of the three seed types on different culture media after a 3-week incubation period are presented in Figures 2 and 3, respectively. Regardless of the seed type, the MGT was not influenced by the different culture media tested. However, an effect of seed type was observed with all culture media. The shortest time for germination (4-5 days) was observed with the embryo axis (EA) followed by the seeds without seed coat (SWtC) which germinated in 8-9 days while the seeds with coat (SWC) have taken between 10 and 14 days to germinate (Figure 2). Compared to the embryonic axis, the time taken by the water to cross the barrier of the integument and to hydrate the cotyledons to initiate the physiological process of germination could explain the delay in germination observed with seeds with or without seed coat. Similarly, [28] demonstrated in a study involving* Uapaca kirkiana* Müll. Arg. that the presence of hard outer seed coat layers delays seed germination due to impermeability and restriction of radical emergence. Similar periods required to achieve* in vitro* germination by EA, SWtC, and SWC, respectively, have already been reported in Bambara groundnut by previous authors [16, 17].

After three weeks of cultivation, no significant difference in the germination rate was observed among the culture media including the control regardless of the type of seed.

This lack of significant difference between basal media and the control containing only agar suggested that macro- and microelements were not necessary for germination in Bambara groundnut and, thus, the success of seed germination was mainly related to water availability. This finding is consistent with the observations reported on the seed germination of* Withania coagulans* Dunal by [29]. Moreover, on the same medium, the germination rate largely varied among the seed types (Figure 3).

Indeed, the germination rates recorded with EA and the seeds without seed coat (SWtC) were similar but significantly higher than the rate obtained with SWC. Obviously, because of the removal of integument, the access to water in SWtC and the EA was more important than in SWC. The mechanical restriction by the seed coat is the reason for the lowest rate of germination obtained with SWC in this species. On the other hand, continuous supply of water is needed to start and complete germination [30].

Studies have also shown that the hard seed coat renders the seeds impermeable to water and oxygen needed for germination process [31]. Higher percentage seed germination was achieved when outer and inner seed coat layers were removed completely [32]. Unlike our results, [17] reported that EA explant gave the highest germination rate among the three explant types throughout the period of culturing.

The mean values of growth parameters of plantlets obtained from the three explants types on different basal media after culturing for four weeks are presented in Table 1. Significant differences in performance/development were observed among plantlets derived from embryo axis explants and those from seeds either with or without coat. The lowest growth performances were observed with the plants grown on the control medium containing no inorganic salts. This result shows that the mineral salts are essential for plant growth after the germination phase and, thus, it is apparent that the nutritional requirements for initiation of germination in Bambara groundnut are considerably different from those required for optimum growth of plantlets.

On the control medium, no significant difference was observed in the development of plants grown from seeds with or without coat. However, the growth of these plants was higher than that of developed plants from the embryonic axis.

This relation was also expressed on basal media containing macro- and microelements. This might be due to adequate nutrient reserves stored in the cotyledon of the SWC and the SWtC with the embryonic axis explants having little stored nutrient reserves. The authors of [17] made similar observations in their studies on Bambara groundnut seeds types. Furthermore, among the five culture media tested, the highest size of the seedling (10.48 cm) and the most important root length (21.51 cm) were observed with the plants developed from seed without coat on MS basal medium.

All tested media contain mineral salts that vary not only in their concentrations but also in their available forms.

The media used in this present study were different from one another in their chemical composition. The distinguishing feature of the MS inorganic salts is their high content of nitrate, potassium, and ammonium in comparison to other salt formulations. MS medium is highly enriched with macro- and microelements and the inorganic salts in this medium were enough to support the maximum growth of the plant. The concentration and the quality of nitrogen in MS medium may be the reason of prolific growth obtained with plant derived seed types incubated on this medium. Indeed, nitrogen is supplied to medium in inorganic form as the anion NO_3_^−^ or the cation NH_4_^+^. The author of [33] found that the ammoniated form of nitrogen is more appropriate than the nitrate form and reported the fastest growth of* Dactylorhiza* species seedlings at 50–100 mg·L^−1^ NH_4_NO_3_. It was also earlier reported by [34] that an efficient concentration of organic and inorganic nitrogen sources can promote the growth of explants. In addition, better plant growth from embryonic axis was observed on MS medium compared to other basal media in* Juglans regia* L. [35].

### 4.2. Effect of MS Medium Strengths on the Growth of Seedlings Developed from the Embryonic Axis and the Seeds without Seed Coat

On the different strengths of MS basal medium used, a significant difference was recorded for growth parameters when comparing the plantlets derived from EA and SWtC (Table 2).

Regardless of the strengths of MS medium, plantlet height, root length, and the biomass were highest with the plants developed from SWtC.

No significant difference was noticed between the three strengths of MS basal medium in terms of number of leaves, root length, and biomass of plantlets derived from both EA and SWtC. But a significant reduction in plantlet height was observed on 1/4 MS. This result shows that a very low amount of macro- and microelements is not effective for plantlet growth. Half strength of MS gave satisfactory results for Bambara groundnut seedling development suggesting that an adjustment can be performed from the full composition of MS basal medium without any significant reduction in plantlet growth. Thus, half MS was selected as the culture medium for the subsequent assessment of carbohydrate treatments.

### 4.3. Effect of Carbohydrate Sources

Plants growing under tissue culture conditions are semiautotrophic [36] and leaves formed during* in vitro* growth may never attain photosynthetic competence [37]. Moreover, plantlets growing under* in vitro* conditions have limited accessibility to CO_2_ inside the vessel [36]. Therefore, sugar is supplemented as a carbon source to maintain an adequate supply of carbon source for* in vitro* multiplication and growth of plant cell, tissue, and organs or whole plantlets. Continuous supply of carbohydrates to plants cultured* in vitro* is essential because the photosynthetic activity of* in vitro*-grown tissues is usually low. These compounds are also necessary as osmotic agents in the culture media. Hence, sugars have a potential effect on the physiology, growth, and differentiation of cells [38]. Therefore, the optimal carbon source needs to be considered. Thus, different sugars such as glucose, fructose, and sucrose at 3% (w/v) were incorporated into the basal medium 1/2 MS.

After four weeks of cultivation, the growth of the plants developed from EA was recorded and presented in Table 3.

Plants grew in the presence of sugar in the medium. The kind of sugar (sucrose, glucose, or fructose) did not seem to have a significant effect on the number of leaves, the root length, and the plant biomass. Similar findings have also been reported in* Arabidopsis thaliana* (L.) Heynh. by [39]. However, among the three types of sugars, the highest plant height (7.94 cm) was observed on medium containing 3% sucrose. The positive effects of sucrose on growth of explants under* in vitro* condition are linked with its high solubility in water, its electrical neutrality, and its lack of inhibitory effect on the majority of biochemical processes [40].

This positive effect of sucrose resulted in its wide use in tissue culture as a carbon source [41, 42]. Supplementation of sucrose in growth medium meets the energy demands for growth and physiological function [36].

### 4.4. Effect of Different Concentrations of Sucrose

Development of* in vitro* plantlets derived from EA was further investigated to study the effect of different concentrations of sucrose, 1, 2, 3, 4, 5, and 6% (w/v), on plant growth. The results obtained after four weeks of culture are recorded in Table 4.

Increasing the sugar concentration from 1 to 3% has a visibly stronger influence on plant height and biomass production. But, above the concentration 3% sucrose, no significant difference was observed. Moreover, among the different concentrations of sucrose tested, a nonsignificant difference was recorded for the number of leaves and the root length. From these results, a 3% sucrose concentration in the basal medium seems to be sufficient for normal plant growth. Sucrose is the most widely used carbon source in most of the plant species, as it is the main sugar translocated in the phloem [19].

As a carbon source, sucrose supports growth of plant cells in culture [43]. A sucrose concentration of 1–5% is generally used for* in vitro* tissue culture, since it is also synthesized naturally by the tissue [44]. For tissue culture, workers generally use 3% sucrose in the medium as per recommendation of [21]. Besides serving as energy source, it also provides the carbon precursors for structural and functional components [45].

## 5. Conclusion

The overall objective of this investigation was to define the optimal conditions for* in vitro* seed germination and plant growth of Bambara groundnut. The main results showed that the composition of the germination medium did not influence the germination capacity of different types of seeds used in Bambara. Germination occurs faster when the embryonic axis is used as a seed source. The best seedling growth is observed with the seeds without coat followed by the embryonic axis on half MS medium containing 3% sucrose. This established protocol would provide sufficient materials as source of explants for initiating in Bambara groundnut different types of tissue culture.

## Figures and Tables

**Table 1:** Growth performance of Bambara groundnut *in vitro* plantlets derived from three explant types on different basal media after four weeks of cultivation.

Media culture	Explant types	Plantlets height (cm)	Number of leaves	Root length (cm)	Biomass (g)
Control	EA	1.93 ± 0.36^d^	2.40 ± 0.24^b^	2.40 ± 0.37^e^	0.01 ± 0.00^d^
	SWtC	2.80 ± 0.30^cd^	2.17 ± 0.10^b^	7.30 ± 0.82^d^	0.11 ± 0.015^b^
	SWC	3.76 ± 0.08^cd^	2.25 ± 0.14^b^	8.07 ± 0.04^d^	0.12 ± 0.001^b^

MS	EA	7.53 ± 0.13^b^	4.88 ± 0.31^ab^	6.70 ± 0.21^d^	0.04 ± 0.003^c^
	SWtC	10.48 ± 0.78^a^	6.99 ± 1.01^a^	21.51 ± 3.02^a^	0.26 ± 0.01^a^
	SWC	7.50 ± 0.7^b^	6.00 ± 0.70^a^	16.59 ± 2.48^b^	0.24 ± 0.007^a^

McCown	EA	5.29 ± 1.20^bc^	3.72 ± 0.55^ab^	3.88 ± 0.84^e^	0.028 ± 0.006^d^
	SWtC	7.17 ± 0.11^b^	6.64 ± 1.57^a^	10.65 ± 3.44^c^	0.25 ± 0.03^a^
	SWC	5.64 ± 0.56^bc^	4.26 ± 0.93^ab^	12.04 ± 0.70^bc^	0.22 ± 0.002^a^

B5	EA	6.99 ± 0.77^b^	4.66 ± 0.33^ab^	4.78 ± 0.42^de^	0.04 ± 0.002^c^
	SWtC	6.23 ± 0.7^bc^	4.67 ± 0.54^ab^	11.98 ± 4.59^c^	0.25 ± 0.02^a^
	SWC	5.76 ± 1.01^bc^	5.55 ± 0.98^ab^	10.95 ± 1.73^c^	0.13 ± 0.009^a^

SH	EA	5.22 ± 1.02^bc^	3.98 ± 0.22^ab^	5.00 ± 0.64^d^	0.03 ± 0.002^c^
	SWtC	7.08 ± 0.60^b^	5.12 ± 0.59^ab^	17.68 ± 3.29^b^	0.22 ± 0.004^a^
	SWC	7.10 ± 1.39^b^	4.90 ± 0.55^ab^	16.15 ± 4.59^b^	0.21 ± 0.001^a^

CHU	EA	5.75 ± 0.47^bc^	4.73 ± 0.39^ab^	3.87 ± 0.06^e^	0.03 ± 0.001^c^
	SWtC	5.80 ± 0.74^bc^	3.66 ± 0.50^ab^	9.55 ± 2.82^c^	0.21 ± 0.02^a^
	SWC	6.20 ± 1.48^bc^	5.16 ± 0.76^ab^	10.81 ± 3.12^c^	0.19 ± 0.03^a^

In the same column, the numbers followed by the same letter are statistically identical to the 5% threshold (Newman-Keuls test) (average ± standard error).

**Table 2:** Growth performance of Bambara groundnut *in vitro* plantlets derived from two explant types on different strengths of MS basal medium after four weeks of cultivation.

Media culture	Explant types	Plantlet height (cm)	Number of leaves	Root length (cm)	Biomass (g)
MS	EA	8.12 ± 0.47^b^	5.70 ± 1.06^b^	6.57 ± 0.23^b^	0.06 ± 0.01^b^
	SWtC	10.72 ± 0.15^a^	7.38 ± 0.52^a^	20.99 ± 0.54^a^	0.51 ± 0.015^a^

1/2 MS	EA	8.13 ± 0.78^b^	5.57 ± 0.81^b^	6.23 ± 0.57^b^	0.05 ± 0.003^b^
	SWtC	10.56 ± 0.84^a^	6.97 ± 0.52^ab^	19.87 ± 1.43^a^	0.51 ± 0.01^a^

1/4 MS	EA	4.96 ± 0.04^c^	5.11 ± 0.29^b^	5.68 ± 0.74^b^	0.02 ± 0.006^c^
	SWtC	8.01 ± 0.04^b^	6.11 ± 0.44^ab^	18.16 ± 1.11^a^	0.38 ± 0.03^a^

In the same column, the numbers followed by the same letter are statistically identical to the 5% threshold (Newman-Keuls test) (average ± standard error).

**Table 3:** Bambara groundnut plantlets growth on 1/2 MS containing glucose, sucrose, and fructose.

Carbohydrates	Plantlet height (cm)	Number of leaves	Root length (cm)	Biomass (g)
Glucose	5.30 ± 0.21^b^	4.90 ± 0.43^a^	6.69 ± 1.14^a^	0.024 ± 0.01^a^
Fructose	4.27 ± 0.33^b^	4.18 ± 0.09^a^	5.50 ± 1.49^a^	0.015 ± 0.001^a^
Sucrose	7.94 ± 0.57^a^	5.35 ± 0.18^a^	5.40 ± 0.65^a^	0.04 ± 0.004^a^

In the same column, the numbers followed by the same letter are statistically identical to the 5% threshold (Newman-Keuls test) (average ± standard error).

**Table 4:** Mean values of growth parameters of Bambara groundnut plantlets on 1/2 MS containing different concentrations of sucrose.

Sucrose concentration (%)	Plant height (cm)	Number of leaves	Root length (cm)	Biomass (g)
1	2.08 ± 0.45^c^	3.63 ± 0.18^a^	4.76 ± 0.86^a^	0.011 ± 0.000^c^
2	4.03 ± 0.34^b^	4.14 ± 0.09^a^	5.97 ± 1.21^a^	0.023 ± 0.001^b^
3	6.99 ± 0.18^a^	4.47 ± 0.35^a^	5.35 ± 0.50^a^	0.031 ± 0.002^ab^
4	7.65 ± 0.71^a^	5.03 ± 0.54^a^	5.87 ± 0.23^a^	0.043 ± 0.000^a^
5	7.64 ± 0.10^a^	5.14 ± 0.19^a^	5.56 ± 0.56^a^	0.063 ± 0.002^a^
6	7.90 ± 0.53^a^	4.68 ± 0.22^a^	5.38 ± 0.36^a^	0.049 ± 0.005^a^

In the same column, the numbers followed by the same letter are statistically identical to the 5% threshold (Newman-Keuls test) (average ± standard error).
